# The Hermit Crab

**Published:** 1919-03-29

**Authors:** 


### HUMAN TEMPERAMENTS.

### The Hermit Crab.

THE hermit crab is a common object of the sea shore. Other crabs are completely enveloped in impenetrable armour, and roam boldly and freely at large, actively searching for food, and living a free, independent, and adventurous life. They possess efficient weapons of offence, and when threatened they show fight, and can give a good account of themselves. The hermit crab is a caricature of a crab. He is deformed, lopsided, and, as it were, only half finished. His forbears were timid creatures. They shrank from the rough-and-tumble of life, and sought refuge in holes and corners where they were comparatively secure from attack. They had not the pluck to fight and strive for their living, but retreated backwards into some secure hole, from which they protruded only their claws and feelers, and with these they picked up such morsels as came in their way. Their favourite refuge was the strong shell of a whelk, which is scarcely vulnerable to any power less than that of a steam-hammer, and as steam-hammers are rare in the sea, they lived secure. The consequence was that their form and structure became adapted to their mode of life. As they mistrusted the protection of their natural armour, and reinforced it by the invulnerable shell of the whelk, Nature deprived them of the armour that they refused to trust in, and left their bodies soft, sensitive, and unprotected. They are covered by nothing but a thin skin, except as to their frontal surface, and they are in the utmost misery and trepidation until they can ensconce their flabby posteriors in the safe hiding-place of a secure retreat, constructed by some other animal for its own security. From the door of this refuge the hermit crab looks out upon a world that he has not the courage or enterprise to mingle with, and takes thankfully such scraps of nourishment as are floated in his way. The menace of danger makes him shrink deeper and deeper into the security of his retreat. Here he lives a vegetative life, without activity, without enterprise, without adventure, the only crisis in his life being the rare occasion on which he ventures to exchange the shell in which he has sought refuge for a larger or more comfortable one. His highest ambition is to live in a larger shell. His chief endeavour is to pass unnoticed. Such is the hermit crab.

The hermit crab has his human counterpart. There are men who, like the common crab, are equipped at all points for the battle of life. They are clothed in the complete armour of self-confidence. They roam the world with enterprise and courage, have all their faculties symmetrically developed, are prepared to encounter all comers and any fortune, fight a good fight, and carve out their own destiny. They stand upon their own legs, go their own way, put up with good fortune or ill fortune, and seek no protection but that of their own capable hands. In contrast with these are the human hermit crabs. These have not the self-reliance of their more active brethren. They have not the activity, the enterprise, the courage, the ability of their better equipped relatives, nor have they the tough integument that can take a blow and be none the worse for it. Their skin is thin, delicate, and vulnerable, and it is highly sensitive. They are so sensitive that they shrink from the rough-and-tumble of active life, from the strife of making a living, from competition, from uncertainty, from risk. "Give me," says the hermit crab, "security. Never mind how small my pittance, so that it is but secure. Not the magnitude but the certainty of my income is what I yearn for. Above all, let me see the prospect of a settled pension at the end of my career. That is my beacon. That is my guiding star. A secure income, to be succeeded by a pension, that is my utmost aspiration; and while I am earning my income, let me not be molested. Let no man find fault with me, for I cannot bear the breath of criticism, and publicity is death to me. Give me the staid invulnerable whelk shell of a Government office, into which I can poke my sensitive posterior, and feel that it is secure from the stings of malice and the kicks of contempt. Relieve me of responsibility, for I am not strong enough to bear it. Let me have a champion to afford me the protection that I cannot provide for myself. Give me orders to carry out, and I will perform them faithfully and to the letter, but do not expect initiative from me, for I have none. Teach me one way of doing things, and I will follow it faithfully to my life's end, or at any rate till I retire upon my pension, without varying by a hair's breadth. A vegetative life for me!"

Such is the hermit crab. A timid, shrinking, sensitive creature, whose craving for security carries him inevitably into a Government or municipal office, or into some other position of security in which the qualities desired are not so much initiative as obedience to orders and adherence to routine. In the complicated organisation of modern society he has his uses. We cannot all be Chinese Gordons or Richard Burtons, and it would be a queer world that was made up exclusively of such men. Unless some men had initiative and enterprise the world would stagnate; but there is much routine work to be done, and the hermit crabs are the men to do it. We must have some men who can be trusted to carry out orders, to fill up forms, to compile returns, to make entries in books, and to do the drudgery; and men of enterprise and initiative cannot do work of this description half as well as the hermit crab, and would be wasted on it if they could.

But the hermit crab has the defects of his qualities. He is a creature of routine, and resents any disturbance of his routine. No disturbance of his routine ever comes from within his office, for it is

**Human Temperaments**—(*continued*).

peopled by other hermit crabs, who share in common his abhorrence of innovation; but outside the office the world goes on. Evolution proceeds; changes are made; progress is achieved; and a time at length comes when the advance can proceed no farther without some change in the law, or without the co-operation, or at least the consent, of some Government Department. To all such change the hermit crab opposes a stubborn, if silent, obstruction. To adopt a change, to sanction a change, is to incur a certain measure of responsibility for the change, and this responsibility he will not incur if he can help it. To oppose the change actively would again incur responsibility. It may be that the change will be beneficial, and if he had actively opposed it he would, when it was eventually adopted, find himself saddled with the responsibility of having opposed it. This would never do; so he does not oppose it actively, but meets it with all the devices of delay and evasion in which he is an adept. If he can only delay long enough, it may be that the innovation will be proved in some other field over which he has no control, and establish itself beyond doubt and beyond question as beneficial. Then he will incur no responsibility by adopting or sanctioning it. Then he will sanction it readily enough; and more than this, if the innovation is so manifestly and undeniably beneficial that it can no longer be doubted, he will, if he can, take to himself, or rather to his office, the credit of having originated it. He does not claim credit for himself. Whatever his faults, disloyalty is not among them. His first care is his own security; but, next to that, and not far short of it, he cherishes the credit of his office; and he will strive for the advantage of his office by means that he would never employ for his own benefit.

The crustacean hermit crab is not wholly vegetative. He has, as we have seen, his ambitions. In time he outgrows his whelk shell and seeks a larger one; and so it is with his human imitator. The aspiration of his life is his retiring pension, and he seldom takes into account that by the time he is able to claim it he will have become so subdued to the routine of his office that beyond it he has no interest and no motive for living. When he can no longer make returns, concoct minutes, make entries in books, and tie up papers, his interest in life is gone, and his pension yields him no enjoyment. The long awaited leisure and independence that have been the aspiration of his life are spent in docketing his own private papers, obstructing innovations in his club, and bemoaning the innovations that he cannot prevent.

Fine specimens of the hermit crab are to be found in Government offices, but of course they are not restricted to Government offices. When the crustacean hermit crab cannot find a suitable whelk shell, he will thrust his sensitive posterior into a shell of another sort; and as fine specimens of the human hermit crab are to be found among the headmasters of public schools, among University professors, among the officials of public asylums, and in other snug berths, as in any Government office; but the Government office is his whelk shell, and it is here that he is to be found in greatest numbers, even if not always in greatest perfection.

